# Optimization of deep learning–based denoising for arterial spin labeling: Effects of averaging and training strategies

**DOI:** 10.1002/mrm.70013

**Published:** 2025-08-05

**Authors:** Jia Guo, Arun Sharma, Greg Zaharchuk, Hossein Rahimzadeh, Naveed Ilyas

**Affiliations:** ^1^ Department of Bioengineering University of California Riverside Riverside California USA; ^2^ Department of Electrical Engineering University of California Riverside Riverside California USA; ^3^ Department of Radiology Stanford University Stanford California USA

**Keywords:** arterial spin labeling, averaging, convolutional neural network, deep learning, denoising

## Abstract

**Purpose:**

Systematic study of the effects of averaging and other relevant training strategies in deep learning (DL)–based denoising is required to optimize such processing pipelines for improving the quality of arterial spin labeling (ASL) images.

**Methods:**

Different averaging strategies, including windowed and interleaved averaging methods, and different levels of averaging before and after convolutional neural network–based and transformer‐based denoising were studied. The experiments were performed on 152 single‐delay ASL scans from 152 subjects, including pulsed and pseudo‐continuous ASL acquisitions. Four‐fold cross‐validation was implemented in all experiments. The effect of including calibration scans (M_0_) was studied and compared across images of different levels of signal‐to‐noise ratio (SNR). The generalizability of DL denoising was examined in experiments using low‐SNR ground truth in training. The results were assessed using image‐quality metrics including structural similarity, peak SNR, and normalized mean absolute error.

**Results:**

Including M_0_ was almost always beneficial, with a dependence on the SNR of the input ASL images. Windowed averaging outperformed interleaved averaging, supporting the practice of reducing scan time. Averaging of ASL images before DL denoising was more advantageous than averaging after. Matching the SNR levels of the images in training and inferencing was important for optimal performance. These findings were consistent across convolutional neural network–based and transformer‐based models. The generalizability of DL‐based denoising was confirmed, and its capability to reduce artifacts was observed.

**Conclusion:**

This study supports the use of DL‐based denoising in improving the image quality of ASL and reducing scan time and provides insights to help optimize DL‐denoising pipelines.

## 1 INTRODUCTION

Arterial spin labeling (ASL) is an MRI technique for quantifying regional perfusion noninvasively^1^ using blood water as an endogenous tracer. In a typical ASL experiment, two types of images (i.e., label and control) are acquired with the arterial magnetization labeled (either by saturation or inversion) or unperturbed, respectively. A period of wait time, referred to as postlabeling delay,^2^ is typically required to allow the arterial blood to reach the tissue/capillaries before image acquisition. After subtracting the label and control images, the tissue signal is canceled out, and the signal difference, or ASL signal, is proportional to the amount of arterial blood delivered during the measurement. Recent advances in ASL^3^, ^4^, ^5^ promote its adoption in various clinical applications^6^, ^7^ using standard recommended imaging protocols,^3^ especially with pseudo‐continuous ASL (PCASL).^8^

One of the major challenges in ASL is the low signal‐to‐noise ratio (SNR), because only a small amount of blood is delivered per unit of time. Even in the brain, which is a highly perfused organ due to high metabolic demands, only about 1% of the volume is replaced by freshly delivered arterial blood, equivalent to a 1% MR signal change. As a result, when the tissue signal is subtracted out, its fluctuations remain and scale with the tissue signal before subtraction, leading to an SNR‐challenging situation. ASL acquisition is usually repeated several times for averaging, which requires a relatively long scan time (e.g., 3–5 min) to reach an acceptable SNR level. In addition, background suppression^9^ is often used to reduce the tissue signals for improved SNR.

In recent years, deep learning (DL) techniques have been introduced to remove the noise of various sources by learning the noise statistics in real‐world ASL data,^10^, ^11^, ^12^, ^13^, ^14^ including the models based on convolutional neural network (CNN) such as U‐Net^12^, ^15^ and dilated wide activation network (DWAN),^11^ and vision transformer (ViT)^16^–based models such as image restoration with hierarchical vision transformer using shifted windows (SwinIR)^17^, ^18^ and image processing transformer.^19^ When compared with conventional denoising techniques, these DL methods have shown improved results^12^, ^14^ and hold great promise for clinical applications. Although these existing DL‐based ASL denoising studies are focused primarily on comparing the performance of different DL architectures, optimization of the overall processing pipeline has not been systematically investigated.

In contrast to most images acquired with other MRI techniques, one of the distinctive features of ASL data is that they typically require a high number of repetitions. Such rich dynamics of the ASL data should allow more flexibility in how the denoising processing pipeline can be implemented. In this study, we systematically examined the effects of data averaging, including additional contrast such as the calibration scan (M_0_) and other training strategies. This enables us to gain a better understanding of DL‐based denoising for ASL and provides useful information to guide its optimization, such as the optimal training or acquisition strategy, given a certain amount of scan time. In addition, we also evaluated the generalizability of DL‐based denoising in ASL, which was assumed by previous studies but has yet to be experimentally verified.

## 2 METHODS

### 2.1 ASL denoising pipeline

**FIGURE 1:**
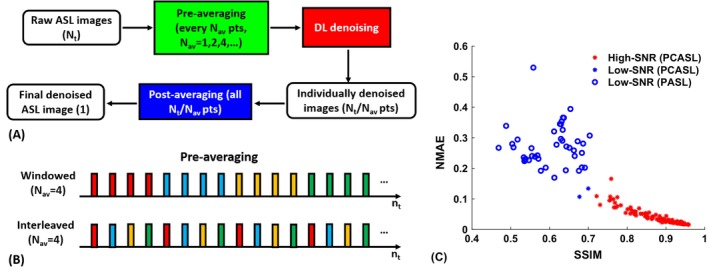
(A) The overall deep learning (DL)–based denoising pipeline. (B) Schematics showing the windowed and interleaved averaging approaches used in the pre‐averaging step, for example, the time points of the same color will be averaged. (C) Dichotomization of high–signal‐to‐noise ratio (SNR) and low‐SNR arterial spin labeling (ASL) data based on the structural similarity index (SSIM) and normalized mean absolute error (NMAE) of the ASL images at the individual time point level. PASL, pulsed ASL; PCASL, pseudo‐continuous ASL.

The overall denoising processing for ASL data is shown in Figure 1A. First, the raw ASL images are generated after subtraction, resulting in N_t_ time points as the input; then, every N_av_ time points are averaged in the pre‐averaging step to produce ASL images of different levels of SNR. These N_t_/N_av_ images are used to train different DL denoising models and produce corresponding individually denoised images in the testing/referencing phase. The DL‐denoised images will be further averaged in the post‐averaging step, to produce the final ASL image. Note that a DL model trained with one number of N_av_ can be applied to ASL images averaged by a different N_av_.

### 2.2 Data set and preprocessing

Most of the ASL data with individual time points were collected from publicly open data sets from OpenNeuro (openneuro.org), consisting of six data sets^20^, ^21^, ^22^ from healthy subjects acquired with PCASL^8^ labeling and one data set^23^ from healthy subjects with pulsed ASL (PASL) using proximal inversion with a control for off‐resonance effects (PICORE)^24^ labeling. In addition, another dataset^25^ from healthy subjects and epilepsy patients using PICORE labeling (shared by Dr. Nazem‐Zadeh) was also included. All the data used in this study were anonymized, and informed consent was collected from all individual participants as reported in each original study. Motion correction was applied on the raw ASL images using *FSL*.^26^ After excluding scans with severe residual motion artifacts and repeated measurements from the same subjects, a data set consisting of 152 single‐delay ASL scans (110 PCASL and 42 PASL) from 152 different subjects, with corresponding M_0_ images, was used; details are summarized in Table 1. These ASL scans with different labeling methods, cohorts, SNR, acquisition time, MRI scanners, and scanning conditions (e.g., two‐dimensional [2D] vs. three‐dimensional [3D] and single‐band vs. multiband) were deliberately included and mixed, as including variation in the data set has been shown to improve the robustness and generalizability of DL models while reducing potential bias.^27^ This also helped increase the size of available data sets, which should further improve the generalizability. To maximize the utility of the data and be consistent with typical acquisition time in most ASL protocols (˜4 min), all the images were resampled into 64 × 64 × 32 using 3D linear interpolation, and the first 32 time points of all scans were used. This data‐set preparation was performed in *MATLAB* (2023b; MathWorks, Natick, MA, USA).

**TABLE 1:** Details of the arterial spin labeling (ASL) data sets used in this study, after excluding scans with obvious motion artifacts. Only the scans with single‐shot readout were included. For Data Sets 4 and 5, the first echo was used. For Data Set 7, the scans under the hormone absent condition were used.

Dataset	1	2	3	4	5	6	7	8
Vendor, scanner	Siemens, Trio	Siemens, Prisma	Siemens, Prisma	GE, MR750	GE, MR750	Siemens, Trio	Siemens, Prisma	Siemens, Prisma
Cohort	Healthy	Healthy	Healthy	Healthy	Healthy	Healthy	Healthy	Healthy (10) & epilepsy (14)
No. subjects	5 (4 males)	4 (2 males)	18 (6 males)	13 (6 males)	7 (4 males)	63 (28 males)	18 (0 male)	24 (13 males)
Age (yo)	27–55	21–65	61–93	20–50	23–58	19–84	18–28	16–41
Labeling method	PCASL	PCASL	PCASL	PCASL	PCASL	PCASL	PASL (PICORE)	PASL (PICORE)
LD/PLD (ms)	1500/1500	1800/1800	1500/1500	1500/1500	1500/1000	1600/1500	700/1100	700/1100
TR (s)	4	4.5	4	3.5	3.5	3.5	4	4.1
Readout	3D, RARE, spiral	3D, RARE, spiral	3D, RARE, spiral	Multiband 2D, GRE, EPI	Multiband 2D, GRE, EPI	3D, GRASE, EPI	2D, GRE, EPI	2D, GRE, EPI
Resolution (mm^3^)	3.8 × 3.8 × 3.8	3.8 × 3.8 × 3.8	3.8 × 3.8 × 3.8	3.0 × 3.0 × 3.0	3.0 × 3.0 × 3.0	4.0 × 3.9 × 7.0	3.0 × 3.0 × 5.0	3.5 × 3.5 × 4.0
Matrix size	64 × 64 × 36	64 × 64 × 34	64 × 64 × 34	80 × 80 × 44	80 × 80 × 36	64 × 57 × 16	64 × 64 × 23	64 × 64 × 35
L/C pairs (time pts.)	49	32	40	45	84	50	44	52
OpenNeuro Accession #	ds000234	ds000235	ds000236	ds000254	ds000216	ds000240	ds003151	N/A

Abbreviations: 2D, two‐dimensional; 3D, three‐dimensional; EPI, echo‐planar imaging; GRE, gradient echo; N/A, not available; PASL, pulsed ASL; PCASL, pseudo‐continuous ASL; PICORE, pulsed ASL using proximal inversion with a control for off‐resonance effects; PLD, postlabeling delay; RARE, rapid acquisition with relaxation enhancement; TR, repetition time; yo, year‐old.

### 2.3 DL model

A full 3D U‐net encoder‐decoder architecture with skip connections at different resolution levels and a residual connection at the center was implemented (Figure S1) using *Python* and *Tensorflow*, similar to a previous two‐dimensional implementation.^27^ Along the encoder arm, at each resolution level, three layers of 3D convolution with batch normalization^28^ and leaky rectified linear unit activation function^29^ were used before a max‐pooling layer, which reduces the dimension of the 3D images by half along each dimension. The initial number of the output channels is set to 16 for the first 3D convolution at the highest resolution and then doubles for the first layer of 3D convolution at each reduced resolution. These convolution/pooling blocks were repeated 4 times, resulting in a tensor size of 4 × 4 × 2 × 128 at the central layer, where the residual connection was applied after a three‐layer convolution. Symmetrically along the decoder arm, three layers of 3D convolution were applied before the resolution doubles using a strided convolution with a stride size of 2. Then the up‐scaled tensors were concatenated using skip connections, with the corresponding tensors from the decoder arm before the next up‐scaling block. To avoid potential overfitting, dropout layers^30^ were included with a dropout rate of 0.05. The final activation function was linear after combining all the channels (64 × 64 × 32 × 16) to produce the final denoised images at the resolution of 64 × 64 × 32. When the M_**0**_ images (64 × 64 × 32) were included in the models, they were treated as an additional input channel.

### 2.4 General training and testing details

To balance the generalizability of the model/study and the computation cost, 4‐fold cross‐validation was implemented for any of the experiments performed in this study. For example, the full data set (152 scans) was randomly divided into four subsets after shuffling. In each of the subfold experiments, 38 scans were preserved for testing/inferencing, and 114 scans were used in training, within which 10% was used in the validation step. The testing results from all 152 subjects were then analyzed together. Data augmentation was used in the training phase, including flipping along x and y, in‐plane transpose, random in‐plane shift (x and y), and rotation (−45° to 45°). After the augmentation, the maximal data size was 30 096 × 64 ×64 × 32, including 32 time points and the calibration scan (M_0_).

The ASL images for each subject were normalized by a global scaling factor such that the mean intensity of the 3D volume was 0.1.^27^ The scaling factor was recorded and could be reapplied to denoised ASL images to recover the original intensity levels. This normalization method (i) preserves the quantification capability of the ASL data; (ii) removes potential internal mean shift in the input distribution (i.e., when combined with the batch normalization method and the nonlinearity of the activation functions in DL training, it helps improve the training stability); and (iii) allows direct pooling and comparison of the results across subjects as the cerebral blood flow (CBF) across subjects and the raw ASL signal acquired on different scanners can vary significantly. The same normalization method was applied on the M_0_ images if they were included in the models.

The training and testing were performed on a GPU (NVidia A6000 Ada). A combined loss function was used in training:

$$F_{\text{loss}}=W*\left(1-\text{SSIM}\right)+\left(1-W\right)*\mathrm{MSE}$$

where W=0.5 was from hyperparameter optimization performed on a small subset of the data; SSIM is structural similarity index^31^; and MSE is mean squared error. For each training condition, the maximal number of epochs was 300. Early termination was allowed after 240 epochs if the validation loss was not improved in 20 epochs. An initial learning rate of 0.002 was used with a minimal value of 0.00001 and a dropping rate of 0.5 after 15 epochs without validation loss improvement. The batch size was adjusted according to the training data size and the available GPU memory. The longest training time was about 100 h for each of the experiments with 100% of the time points and M_0_ image, 4‐fold cross‐validation, and a full set of N_av_ (1, 2, 4, 8, and 16). For other conditions, such as without M_0_ or using 50% of the time points, the training time was shortened accordingly.

### 2.5 Experimental design

#### 2.5.1 Effects of including calibration scans (M_0_ )

Previous studies^12^, ^14^, ^27^, ^32^, ^33^ have shown that including additional contrasts such as T_1w_ and T_2w_ anatomical information is advantageous in end‐to‐end DL tasks. In this study, we limited the use of anatomical information to only include the M_0_ images (i) to maximize the method's utility, as almost all ASL scans have M_0_, but other anatomical images (e.g., T_1w_) may not be readily available; and (ii) to minimize the confounding contribution from non‐ASL information in examining the denoising behaviors of DL models. The DL models were trained and tested without and with the M_0_ images included, under different averaging strategies (see subsequently). Then, the results were analyzed at different SNR levels. To more closely study the effect of SNR on denoised image‐quality improvement, the ASL images were also dichotomized into low‐SNR and high‐SNR groups using k‐means clustering on SSIM and normalized mean absolute error (NMAE) of the input images at the single‐time‐point level (Figure 1C) for separate comparisons. The results from this experiment were also used to guide the following experimental design.

#### 2.5.2 Averaging strategies

We performed DL‐based denoising with different commonly used averaging methods to explore their effects on the denoising performance, aiming to identify optimized strategies for ASL data with different qualities. Specifically, the following aspects of the averaging strategies have been studied, and we use N_av_pre_ and N_av_post_ to further differentiate the N_av_ used in the pre‐averaging and postaveraging steps. In performance analyses in experiments described under Section 2.5.2, the averaged ASL images using all time points (N_av_ = 32) were used as the ground truth (GT) (i.e. GT_100%_).

##### Number of averages before DL noising

In this experiment, all time points (N_t_ = 32) were used. As shown in Figure 1A, a set of DL models were trained on input ASL images with N_av_pre_ = 1, 2, 4, 8 and 16, and with GT_100%_. The quality of the denoised images were then analyzed at two stages: (i) on the individually denoised ASL images before postaveraging (i.e., N_av_post_ = 1 for all); and (ii) on those after postaveraging (i.e., with corresponding N_av_post_ = 32, 16, 8, 4 and 2, respectively).

##### Windowed versus interleaved averaging

To gain a better understanding of the effects of averaging strategies and corresponding temporal noise patterns, we further explored two different averaging approaches at the pre‐averaging stage: interleaved and windowed averaging, as shown in Figure 1B, with N_av_pre_ = 2, 4, 8, and 16. Interleaved averaging was studied to test whether including the information of perfusion signal fluctuations spanning a wider range of time is beneficial, although windowed averaging is more reflective of the actual acquisition practice, especially with scan time reduction. Comparing these two would help determine whether and how much reducing scan time in practice, such as with windowed averaging, will compromise the accuracy of perfusion estimation when the observation period is limited. The same windowed or interleaved averaging approach was applied in corresponding training and testing, so that the noise fluctuation patterns were closely matched for valid comparison.

##### Exploring optimal averaging strategies under different SNR scenarios

In this experiment, different SNR/scan time scenarios were simulated in the testing phase, where available ASL data with N_t_ = 1, 2, 4, 8, and 16 as input were studied with different combinations of N_av_pre_ and N_av_post_ using the results from the windowed versus interleaved averaging experiment, where N_av_pre_ * N_av_post_ = N_t_. To minimize potential bias in the analysis results, the quality metrics were averaged across instances at any given combination of N_av_pre_ and N_av_post_. For example, with N_av_pre_ = 2 and N_av_post_ = 4, there were four instances available for averaging, given the total time points of 32. With different N_t_, the optimal N_av_pre_ and the corresponding DL model were identified based on the image quality metrics; and similarly, the optimal combinations of N_av_pre_ and N_av_post_.

#### 2.5.3 Generalizability of DL denoising

Can the DL denoising approach improve ASL image quality beyond that of the ground truth used in training? To answer this, the training data and the “ground truth” image were restricted to a subset of the data (i.e., using 25% and 50% of the total time points, corresponding to GT_25%_ and GT_50%_, or 8 and 16 time points, respectively); in the testing phase, the previous GT images, GT_25%_ and GT_50%_ were used as the input, and then the denoised images were compared with the real GT (GT_100%_) to test whether the DL denoising performance extends beyond the training (i.e., the generalizability of DL‐based denoising).

#### 2.5.4 Contribution of averaging and DL in denoising performance

The image quality after the pre‐averaging, after the DL denoising, and after the postaveraging steps was compared with that of the input images before averaging. The differences (i.e., the image quality improvement [Δ]) at these processing steps were calculated and averaged across time points and subjects to examine their contributions accordingly. By comparing the quality improvement from DL denoising versus that from increasing scan time (more averaging), a more direct and quantitative understanding of the denoising processing may be gained to help us determine optimized acquisition strategies for ASL scans, such as a feasible scan time reduction without significant reductions in image quality.

#### 2.5.5 Validation with a transformer architecture

To verify the major findings in this study, we also adapted the three‐slice pseudo‐3D SwinIR architecture using the recommended setting,^14^ except that the normalization method for the input and the cross validation in training and testing was the same as that used in the 3D U‐net experiments. The results presented subsequently are obtained with the 3D U‐net architecture, unless specified otherwise. Some of the pseudo‐3D SwinIR results can be found in the Supporting Information.

### 2.6 Performance evaluation and statistical analysis

The quality of the ASL images were evaluated using three commonly used metrics, including SSIM, peak SNR (PSNR), and NMAE, using GT_100%_ as the reference, where all the images were normalized as described previously. We did not perform CBF quantification because of the large variability of absolute CBF in different subjects. Instead, NMAE can serve as the indicator for perfusion quantification error, where the percentage perfusion error can be directly calculated (i.e., NMAE is equivalent to a scaled absolute CBF percentage error, given the normalization method applied in this study). All the images were brain‐masked in the result analysis stage only to calculate these metrics, whereas the unmasked images were used in training and testing, including the loss calculation. Brain masking helped better delineate the denoising performance in the brain for a fair comparison. This is because the significant reduction of artifacts and noise outside of the brain, while it improves the visual quality, is less relevant in examining the perfusion patterns in the brain and could favorably bias the performance evaluation for DL denoising.

The Jarque‐Bera test was used to examine the normality of the difference between the corresponding counterparts under comparison. Then, the pairwise t‐test and signed‐rank test were performed for normal and nonnormal distributed results, respectively, with the *p*‐value reported and a significance detection threshold (α) of 0.05, with Bonferroni correction in case of multiple comparisons.

## 3 RESULTS

The data dichotomization results are shown in Figure 1C. All PASL data (42 of 42 scans) were clustered into the low‐SNR group, whereas almost all PCASL data (108 of 110 scans) were in the high‐SNR group, showing generally higher SNR than PASL at the individual time point level, indicated by the SSIM and NMAE metrics.

**FIGURE 2:**
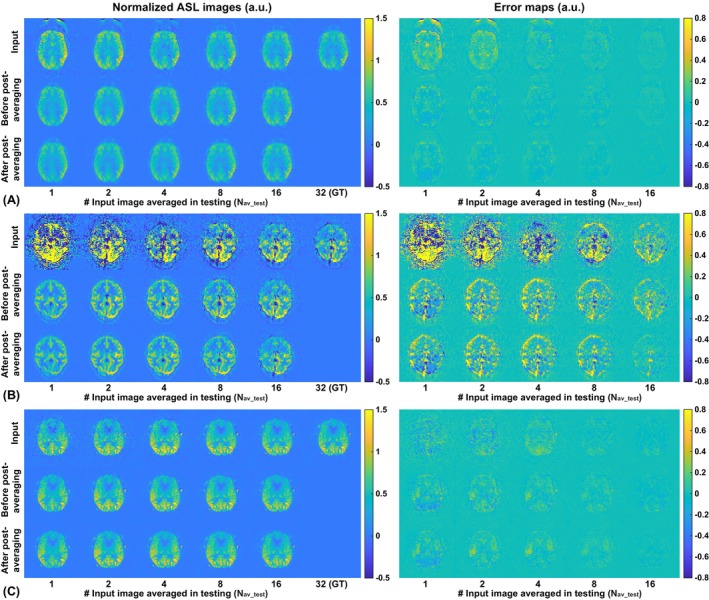
Examples of deep learning (DL)–denoised arterial spin labeling (ASL) images, with the input, ground truth (GT; *first row*), individually denoised (before postaveraging; *middle row*), and postaveraged images (*third row*) shown in the left panels, and the corresponding error maps in the right panels. (A) A representative example, also showing the artifact reduction after DL denoising, especially outside of the brain. (B) Example from the low‐SNR group. (C) Example from the high–SNR group.

Representative denoised ASL images are shown in Figure 2; additional examples can be found in Figures S2–S4. These ASL images were denoised using the DL models trained with a matched number of averages (i.e., $N_{\mathrm{av}\_\mathrm{pre}}=N_{\mathrm{av}\_\text{test}}$) and with M_0_ included. These results demonstrated the quality of DL denoising at different SNR levels, with noise reduction throughout the images and indicated by the error maps. The noise reduction was more remarkable in the low‐SNR images than in the high‐SNR images due to the prevailing presence of the noise. The example in Figure 2A also showcases obvious reduction of artifacts outside of the brain and presumably a similar reduction in the brain. Another interesting observation, as shown in other examples, the real blood flow signals—including those perfused and intravascular signals in the scalp—were well preserved.

**FIGURE 3:**
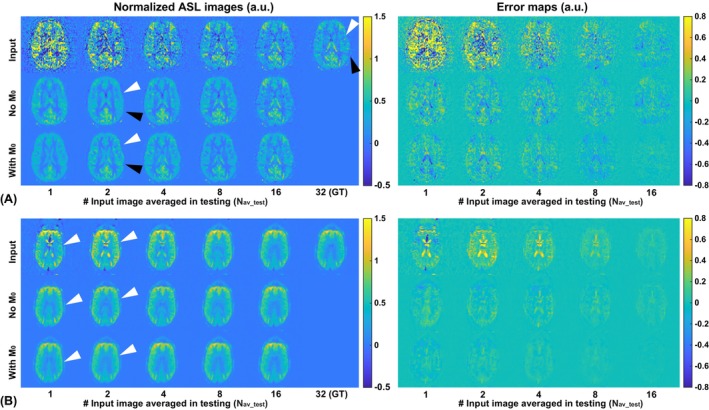
Representative examples of denoised images without and with the calibration scan (M_0_) included in the deep learning model. (A) Example showing more accurate anatomical details and higher resolution (*black and white arrowheads*) with M_0_ included compared to that without. (B) Example showing more accurate anatomical details when M_0_ was included, as well as the artifacts reduction in the ventricles observed in both models without and with M_0_ included (*white arrowheads*). ASL, arterial spin labeling.

**FIGURE 4:**
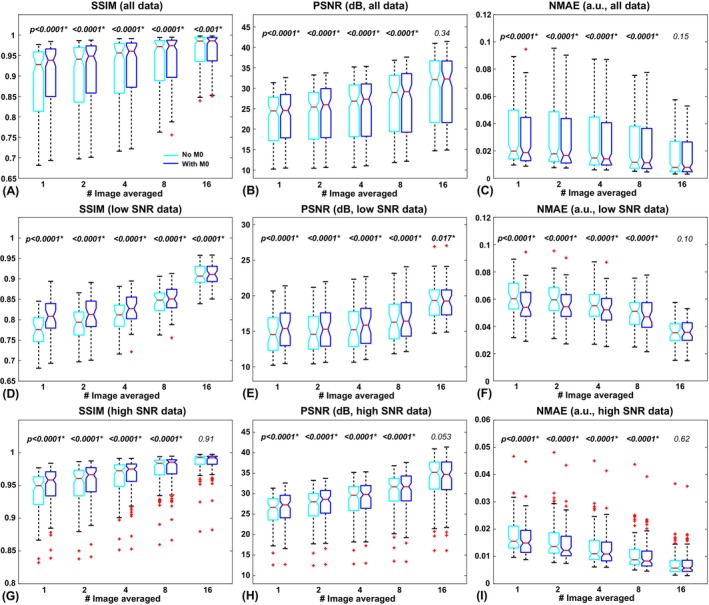
Comparison of averaged image‐quality metrics between deep learning models trained without and with M_0_ included in the model. The numbers of averaging condition were matched in training and testing. (A–C) Averaged image‐quality comparison across all subjects. (D–F) Comparison performed in the low–signal‐to‐noise ratio (SNR) group. (G–I) Comparison performed in the high‐SNR group. The *p*‐values from the statistical tests were reported at the top, with significant differences labeled with asterisks and shown in bold font. NMAE, normalized mean absolute error; PSNR, peak SNR; SSIM, structural similarity index.

Examples of the results obtained without and with including M_0_ are shown in Figure 3. The results of comparison on the image‐quality metrics are shown in Figure 4. With low‐SNR and high‐SNR results pooled, the DL models with M_0_ included showed significantly better performance in SSIM, PSNR, and NMAE when $N_{\mathrm{av}\_\mathrm{pre}}=N_{\mathrm{av}\_\text{test}}=1,2,4\;\text{and}\;8$. When $N_{\mathrm{av}\_\mathrm{pre}}=N_{\mathrm{av}\_\text{test}}=16,$ including M_0_, yielded better SSIM scores but comparable PSNR and NMAE scores when compared with the models without M_0_, a closer examination on the performance in the low‐SNR and the high‐SNR groups showed very similar trends. The improvement (difference) was greater in low‐SNR situations (i.e., in the low‐SNR group or with smaller N_av_) when the SNR of the input images was smaller. For example, when $N_{\mathrm{av}\_\mathrm{pre}}=N_{\mathrm{av}\_\text{test}}=16$, none of the metrics showed significant performance difference in the high‐SNR group, nor did the NMAE of the low‐SNR group. This is consistent with previous studies that including anatomical information, such as M_0_, helps improve the performance of DL models in general. These new results qualitatively and quantitatively showed that the improvement by including M_0_ depends on the SNR of the input ASL images. The lower the SNR, the more likely the DL models would use relevant anatomical information to improve the image quality. When the SNR of ASL images was sufficiently high, including M_0_ in the DL model yielded at least comparable, if not significantly improved, quality compared with that without M_0_. These findings were also verified with the SwinIR transformer architecture (Figure S5).

**FIGURE 5:**
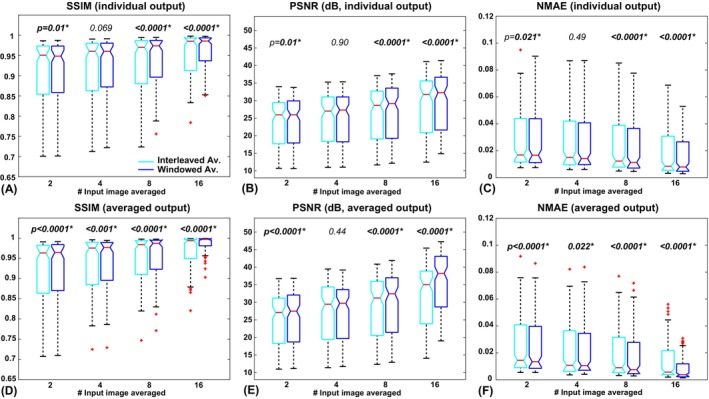
Comparison of the denoising performance between the interleaved and windowed averaging approaches. (A–C) Comparison of the averaged image‐quality metrics after the individually deep learning–denoised step. (D–F) Comparison of the averaged image‐quality metrics after the postaveraging step. Overall, windowed averaging yielded superior performance than interleaved averaging at both processing steps. NMAE, normalized mean absolute error; PSNR, peak signal‐to‐noise ratio; SSIM, structural similarity index.

The results of comparison between the interleaved and windowed averaging methods are shown in Figure 5 for 3D U‐net, and in Figure S6 for pseudo‐3D SwinIR, respectively. At both individually denoised and postaveraged stages, windowed averaging outperformed interleaved averaging at almost all tested N_av_ with both 3D U‐net and pseudo‐3D SwinIR architectures, with only a few exceptions where some of the image‐quality metrics suggested comparable performance between the two. Another observation was that the windowed averaging method outperformed the interleaved averaging methods more with increased N_av_.

**FIGURE 6:**
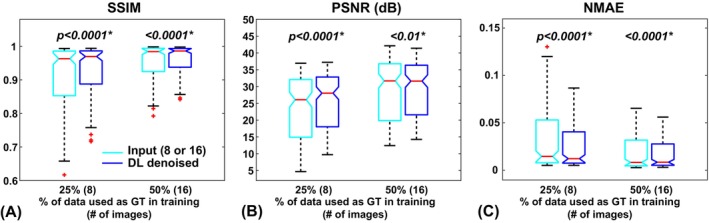
Results of applying deep learning (DL)–denoising models that were trained on low–signal‐to‐noise ratio (SNR) semi–ground truth images (GT_25%_ and GT_50%_, respectively) to these GT_25%_ and GT_50%_ images. Such further improvement demonstrated the generalizability of the DL‐denoising approach (i.e., the capability to learn noise statistics and extend the denoising performance beyond that in the ground truth used in training). NMAE, normalized mean absolute error; PSNR, peak SNR; SSIM, structural similarity index.

Figure 6 presents the results when only a portion of the time points were used in model training (GT_25%_ and GT_50%_). In both cases, when the models were trained and applied on the same partial GTs, the denoising behavior indeed persisted and extended beyond what was used in the training, showing further improved image quality when compared with the real ground truth (GT_100%_). This clearly demonstrates the generalizability of the DL‐based denoising method.

The averaged image‐quality metrics obtained using DL models trained and tested under different N_av_ are summarized in Table 2 for 3D U‐net and in Table S1 for pseudo‐3D SwinIR, respectively. For DL‐denoising itself, the best performance was achieved when N_av_ was matched in training and testing/inferencing. However, when the postaveraging step was included, the best results were achieved using the model trained with the highest number of N_av_ (16) and subsequent postaveraging.

**TABLE 2:** Denoising performance before (i.e., individually denoised) and after the postaveraging step.

		Mean SSIM					Mean PSNR (dB)					Mean NMAE				
Ind. denoised		N_av_test_					N_av_test_					N_av_test_				
		1	2	4	8	16	1	2	4	8	16	1	2	4	8	16
N_av_train_	1	**0.9069**	0.9123	0.9171	0.9210	0.9246	**23.2652**	23.8165	24.2821	24.6512	24.9368	**0.0279**	0.0266	0.0255	0.0246	0.0239
	2	0.9049	**0.9147**	0.9226	0.9288	0.9337	23.1516	**24.1026**	24.9656	25.6891	26.2763	0.0289	**0.0265**	0.0246	0.0231	0.0220
	4	0.8973	0.9126	**0.9252**	0.9355	0.9441	22.5908	23.8425	**25.0612**	26.1632	27.1695	0.0310	0.0274	**0.0244**	0.0219	0.0199
	8	0.8800	0.9027	0.9227	**0.9393**	0.9532	21.4788	23.2245	24.9975	**26.7644**	28.5923	0.0364	0.0302	0.0252	**0.0212**	0.0177
	16	0.8254	0.8615	0.8977	0.9313	**0.9633**	16.6625	19.5168	22.5385	25.6525	**29.4838**	0.0687	0.0492	0.0350	0.0245	**0.0157**

After post‐av.		N_av_test_					N_av_test_					N_av_test_				
		1	2	4	8	16	1	2	4	8	16	1	2	4	8	16
N_av_train_	1	0.9159	0.9189	0.9215	0.9238	0.9259	24.2271	24.5285	24.7559	24.9242	25.0493	0.0257	0.0250	0.0244	0.0240	0.0236
	2	0.9216	0.9265	0.9305	0.9335	0.9357	25.0274	25.6084	26.0536	26.3682	26.5744	0.0246	0.0234	0.0225	0.0219	0.0215
	4	0.9271	0.9341	0.9397	0.9443	0.9480	25.6367	26.3846	27.0003	27.4632	27.7993	0.0234	0.0219	0.0206	0.0196	0.0188
	8	0.9362	0.9439	0.9504	0.9560	0.9605	26.9265	27.9963	28.8870	29.5815	30.0913	0.0210	0.0193	0.0179	0.0166	0.0156
	16	**0.9757**	**0.9801**	**0.9833**	**0.9857**	**0.9878**	**32.8980**	**33.9512**	**34.7535**	**35.3391**	**35.7308**	**0.0114**	**0.0101**	**0.0090**	**0.0082**	**0.0075**

*Note*: For visual clarify purposes, only the mean values are reported, and the best results under each N_av_test_ condition are shown in bold font and shaded cells. Without postaveraging, the best results for each N_av_test_ condition were achieved when the number of averages used in the training model matched that used in testing (i.e., N_av_train_ = N_av_test_); with postaveraging, the best results were obtained with the models trained with the maximal N_av_train_ value of 16 and then averaged.

Abbreviations: NMAE, normalized mean absolute error; post‐av., postaveraging; PSNR, peak signal‐to‐noise ratio; SSIM, structural similarity index.

Varying the portion of time points used was used to simulate and test more realistic scenarios (i.e., reduced scan time, different combinations of pre‐averaging and postaveraging, and different DL denoising models). These averaged image‐quality metrics are summarized in Table 3 for 3D U‐net and in Table S2 for pseudo‐3D SwinIR, respectively. The best performance under each available N_tp_ condition was achieved when a model trained with a matched N_av_ was consistent with the results when all data were available (N_tp_ = 32). Comparing different pre‐averaging and postaveraging combinations, a clear overall trend was observed that the best image quality was achieved with a highest pre‐averaging number that was allowed in a given set of ASL input images, with only a few exceptions. These findings were consistent across the two representative CNN and transformer architectures, demonstrating the validity of such patterns.

**TABLE 3:** Optimized denoising performance with input data of different available time points as input.

		Mean SSIM (N_av_train_)					Mean PSNR (N_av_train_)					Mean NMAE (N_av_train_)				
		Averaging method (N_av_pre_:N_av_post_)					Averaging method (N_av_pre_:N_av_post_)					Averaging method (N_av_pre_:N_av_post_)				
		1:N_tp_	2:(N_tp_/2)	4:(N_tp_/4)	8:(N_tp_/8)	16: (N_tp_/16)	1:N_tp_	2:(N_tp_/2)	4:(N_tp_/4)	8:(N_tp_/8)	16: (N_tp_/16)	1:N_tp_	2:(N_tp_/2)	4:(N_tp_/4)	8:(N_tp_/8)	16: (N_tp_/16)
N_tp_ available	1	**0.9069 (1)**					**23.2652 (1)**					**0.02789 (1)**	0	0	0	0
	2	0.9132 (2)	**0.9147 (2)**				23.9923 (2)	**24.1026 (2)**				0.02680 (2)	**0.02649 (2)**	0	0	0
	4	0.9194 (4)	0.9235 (4)	**0.9252 (4)**			24.9208 (8)	**25.0858 (8)**	25.0612 (4)			0.02534 (4)	0.02463 (4)	**0.02438 (4)**	0	0
	8	0.9342 (16)	0.9340 (16)	0.9373 (8)	**0.9393 (8)**		25.9241 (8)	26.4464 (8)	26.7102 (8)	**26.7644 (8)**		0.02301 (8)	0.02202 (8)	0.02143 (8)	**0.02119 (8)**	0
	16	0.9595 (16)	0.9618 (16)	0.9629 (16)	**0.9634 (16)**	0.9633 (16)	29.1979 (16)	29.4487 (16)	**29.5456 (16)**	29.5393 (16)	29.4838 (16)	0.01647 (16)	0.01587 (16)	0.01562 (16)	**0.01557 (16)**	0.01569 (16)

*Note*: The number of averages in training (N_av_train_) shown in parentheses are the values to achieve the best results across different deep learning models trained with different N_av_train_ values under that experimental condition. For visual clarify purposes, only the mean values are reported. The best results under each Data Availability condition are shown in bold font and shaded cells.

Abbreviations: NMAE, normalized mean absolute error; PSNR, peak signal‐to‐noise ratio; SSIM, structural similarity index.

**FIGURE 7:**
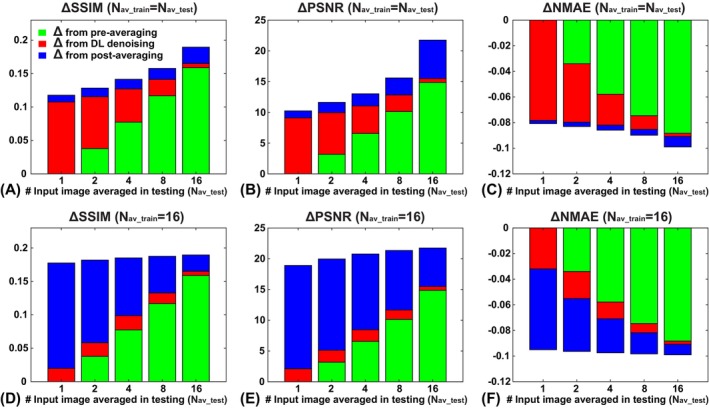
The image‐quality metric improvement (Δ) achieved by different processing steps in the denoising pipeline, using the images at individual time points as the reference, where green bars correspond to the improvement from the pre‐averaging step, red the improvement from the deep learning (DL)–denoising step, and blue the improvement from the postaveraging. (A–C) Results using deep learning models that have matched N_av_ in pre‐averaging in both training and testing (e.g., the results with N_av_test_ = 2 used the model trained with N_av_train_ = 2). (D–F) Results using DL models trained with N_av_train_ = 16 under all testing conditions. These results were obtained with all the time points used (e.g., with GT_100%_) and averaged across all subjects/scans. NMAE, normalized mean absolute error; PSNR, peak signal‐to‐noise ratio; SSIM, structural similarity index.

The corresponding averaged step‐by‐step image‐quality improvements are shown in Figure 7 for 3D U‐net and in Figure S7 for pseudo‐3D SwinIR, respectively. The image‐quality metrics improved as more time points were included in pre‐averaging, as expected. The improvement from DL denoising, however, depended on how the models were trained with the level of pre‐averaging. For example, when a model was applied to the input images with a matched N_av_, the DL‐induced improvement was greater than that with unmatched N_av_, as also demonstrated in Table 2. Overall, a lower N_av_ (or SNR) used in training resulted in a more aggressive DL denoising. On the other hand, the effects of postaveraging showed a different dependence on what DL model was applied. For example, under the same N_av_test_ condition, the less aggressive the DL denoising step, the more improvement from the postaveraging, and vice versa. The overall quality improvement, although bound by how noisy the input images are, favors a processing pipeline with more pre‐averaging and correspondingly less postaveraging, as shown in Figure 7D–F. The same trends were observed when the results were separately analyzed in the low‐SNR and high‐SNR groups, as well as with the models trained with GT_50%_ (Figures S8–S10).

## 4 DISCUSSION

This is the first study to systematically examine the effects of using the temporal dynamics of ASL data to optimize DL‐based denoising methods, with quantitative evaluation on the image‐quality improvement with respect to averaging strategies, scan time reduction, and other training considerations. The results confirmed the benefits of including anatomical information, such as M_0_, and revealed valuable information on optimization of DL‐based denoising processing. These findings were consistent across two different representative DL architectures (i.e., 3D U‐net CNN and pseudo‐3D SwinIR transformer). According to the evidence in the current study, a generally optimized denoising strategy for ASL would require a model trained with data of similar noise levels, and prefer a windowed averaging method and maximal averaging before passing the images to DL denoising. In addition, we verified the generalizability of DL‐based denoising. For example, the DL models were able to learn some noise characteristics that can be generalized to improve the image quality beyond that of the GT used in training.

Including M_0_ in the DL‐based denoising model, as demonstrated by this study and others,^12^, ^14^, ^27^, ^33^ helps improve the image quality by providing additional anatomical reference information, and is recommended whenever available. This study further showed that such anatomical information (M_0_) was used in an adaptive way: When the SNR was low, the DL models relied more on such anatomical information to help improve the image quality as needed, whereas when the SNR was sufficiently high, the DL processing weighted the actual and reliable ASL signals much more, such that including M_0_ did not make a significant difference. However, cautions should be exercised when SNR is very low, whereas relying too much on anatomical information may introduce unwanted bias, especially when the functional information (perfusion) does not match the anatomical information. Similar observation was also reported by Shou et al.^14^ However, we did not observe the large increases of bias when M_0_ was included in this study using both the 3D U‐net and the pseudo‐3D SwinIR architectures, judging from the normalized mean error metric (Figure S11). This may be attributed to the normalization processing implemented in this study, which should help reduce the variation in the overall ASL signal level across scans and subjects, as well as the intensity discrepancy between the ASL and the M_0_ images. In addition, this potential concern could be addressed by including more data with anatomical‐functional mismatch. It is reasonable to expect that including additional anatomical information, such as T_1w_ or T_2w_ images, may be overall beneficial when available, but caution is needed as the contrast of these images may vary dramatically and they should be matched in training and inferencing.

It was unexpected that interleaved averaging yielded inferior results than windowed averaging, as one would expect that including data from wider time spans would help better capture the averaged perfusion, given that the GT was generated using all the time points. It is possible that the ASL signal and noise have different fluctuation patterns at the local and the global levels, and the DL models were more adept in learning the former. This needs further investigation. Nonetheless, our study provides the first true experimental evidence to show that reducing ASL scan time in practice (i.e., with windowed averaging) with DL denoising does not significantly compromise the image quality, especially when compared with a scan of the same amount of images acquired over a longer period of time, under the assumption that the perfusion patterns remain relatively stable during a baseline perfusion scan.

Comparing the image‐quality improvement with different levels of averaging (Figures 7 and S7), a 2‐fold acceleration should be readily achievable with a modest DL denoising (e.g., with N_av_train_ = N_t_/2) without any image quality degradation. A 4‐fold, or even 8‐fold, acceleration seems feasible when more SNR‐matched models are trained and applied, given the CNN‐based and transformer‐based architectures and the availability of training datasets used in this study.

Generally, the DL models trained with lower SNR data demonstrated stronger denoising behaviors, as demonstrated, for example, by less noise outside of the brain in the examples shown in Figures 2 and 3 when the models with low N_av_train_ were applied. This is expected, as the DL training learns to minimize the difference between the input (low‐SNR) and the GT (high‐SNR) ground truth, where a greater amount of noise was to be removed. However, such heavy denoising behavior does not necessarily result in optimal quality improvement, as suggested by the quality metrics reported in Tables 2, 3, S1, and S2. Instead, the results suggested that the optimal DL denoising performance requires a good match of the SNR levels between the images in training and inferencing, highlighting the importance of understanding the denoising behaviors and the application boundaries of trained DL models. This also calls for better designed denoising methods that can be more adaptive. For example, including L1 norm in the loss function may help reduce the blur and improve the anatomical accuracy when applied in low SNR images.

DL denoising showed strong nonlinear effects on the temporal noise patterns, especially in the models trained with low SNR input. With these models, DL denoising significantly improved the image quality at the individual input level, but the postaveraging could not further improve the quality as much as one would expect due to altered noise statistics, thus resulting in overall suboptimal denoising performance. One such example can be found when comparing the improvements in the N_av_test_ = 1 case using DL models with N_av_train_ = 1 and 16 (Figure 7), where a moderate denoising with N_av_train_ = 16 outperformed that with N_av_train_ = 1 with further noise reduction in postaveraging, even though the same number of processed images were available in postaveraging.

Furthermore, when comparing the effects of pre‐averaging and postaveraging in the experiments simulating more realistic scenarios "the experiment simulating different SNRs, the results in Table 3 suggested that pre‐averaging was more beneficial than postaveraging with a given amount of raw ASL data, when a properly trained DL model (i.e., with similar SNR level in training) was applied. The overall quality improvement was optimal with more pre‐averaging to reduce the noise in the input. This is a desired feature, as such a processing strategy can significantly reduce the computation cost with dramatically reduced data size in training DL models.

In addition to noise suppression, DL‐based denoising demonstrated excellent suppression of artifacts. As described earlier, the data analyses focused on evaluation in the masked brain region to avoid potential biases, as the regions outside of the brain are generally less relevant. Nonetheless, the significantly reduced noise outside of the brain improved the visual quality of the images, as shown in Figures 2A and S2, where they were likely caused by some transient motion. Suppression of artifacts within the brain was also observed, as shown in Figure 3B, where the labeling artifacts in the ventricles with small N_av_test_ were satisfactorily suppressed. Interestingly, the noise suppression worked well without including additional anatomical information (M_0_); when M_0_ was included, such suppression worked even better. On the other hand, when the “artifacts” were actually ASL signals, such as intravascular signals caused by insufficient postlabeling delay, DL denoising faithfully preserved such ASL signals, as shown in the example in Figure S3. This is important in clinical applications where such artifacts may carry critical diagnostic information about the vascular health of patients with conditions such as stroke or arteriovenous malformation.

There were limitations in the current study that can be addressed in future investigation. First, only two representative DL architectures (i.e., 3D U‐net CNN and pseudo‐3D SwinIR transformer) were studied. Although it is reasonable to expect that the findings are likely to hold with other DL architectures, we did not examine this explicitly. Second, all ASL raw images were motion‐corrected before subtraction, and the data sets with obvious motion artifacts beyond such correction were excluded. Motion‐induced image‐quality degradation still existed in some scans. Although the results demonstrated excellent reduction of motion‐like artifacts, they were not readily separated from other noise sources such as those of physiological origins. It may be worth further study to improve our understanding of DL's capability of suppressing these artifacts. Third, although including relatively low SNR PASL data can improve the generalizability of DL models, the low SNR in corresponding GT images was not ideal for DL training and may have limited the optimal performance the models can achieve. Also, in addition to studying effects of the number of averages used in training different DL models, grouping the input images based on their SNR levels may help reveal additional and useful patterns of DL denoising; however, this was not performed due to limited data availability in the current study. Finally, all the ASL images used in this study were acquired with single‐shot readouts. ASL images acquired with multishot readouts have far fewer averages/time points where individually subtracted pairs correspond to a longer period of time. It is unclear whether the trends identified here would hold for other multishot readout strategies.

## 5 CONCLUSION

We systematically studied the effects of different averaging and training strategies on the performance of DL‐based denoising for ASL and have shown that the noise patterns of ASL images can be dramatically altered by different DL‐denoising models. The results demonstrated that windowed averaging outperforms interleaved averaging, and averaging of the ASL images before DL denoising is more advantageous than averaging after DL denoising. Including M_0_ images is almost always beneficial and recommended, but the accuracy may be compromised at very low SNR levels. These findings were validated and consistent across different and representative DL models based on CNN and transformer architectures. In addition, the generalizability of DL‐based denoising has been verified. These results further support the application of DL‐based denoising of ASL images in practice, and help provide insights on optimization of such processing pipelines.

## Supporting information


**Figure S1.** A schematic showing the three‐dimensional (3D) U‐net encoder‐decoder architecture used in this study. The numbers of the input channels are 1 and 2 for the models without and with M_0_ included, respectively. The deep learning operations and the dimensions of the tensors are color‐coded, as shown in the legends, and the channel numbers of the tensors after max pooling are shown at the top of the tensor blocks with slightly darker colors. BN, batch normalization; CONV, convolution; ReLU, rectified linear unit.
**Figure S2.** An example showing the deep learning (DL)–denoising processing was able to suppress artifacts throughout the images which were likely caused by motion.
**Figure S3.** Example showing that while the noise and some minor motion artifacts were satisfactorily suppressed by deep learning (DL) noising, the flow‐related artifacts (i.e., intravascular ASL signals) were faithfully preserved.
**Figure S4.** Another example showing the deep learning (DL)–based denoising performance in a low–signal‐to‐noise ratio (SNR) scan. Some regional biases were observed when the DL models were trained and tested with low N_av_ values due to low SNR.
**Figure S5.** Like that shown in Figure 4, except that the results were obtained with the pseudo‐3D (three‐slice) SwinIR transformer architecture. Trends like those using the 3D‐Unet architecture can be clearly observed. 3D, three‐dimensional.
**Figure S6.** Like that shown in Figure 5, except that the results were obtained with the pseudo‐3D (three‐slice) SwinIR transformer architecture. Trends like those using the 3D U‐net architecture can be clearly observed, demonstrating that windowed averaging yielded superior performance than interleaved averaging overall. 3D, three‐dimensional.
**Figure S7.** Like that shown in Figure 7, except that the results were obtained with the pseudo‐3D SwinIR transformer architecture and with all the time points used (e.g., with GT_100%_) and averaged across all subjects/scans. 3D, three‐dimensional.
**Figure S8.** Like that shown in Figure 7, except that the results were obtained with all the time points used (e.g., with GT_100%_) and averaged across the high–signal‐to‐noise ratio (SNR) scans only.
**Figure S9.** Like that shown in Figure 7, except that the results were obtained with all the time points used (e.g., with GT_100%_) and averaged across the low–signal‐to‐noise ratio (SNR) scans only.
**Figure S10.** Like that shown in Figure 7, except that the results were obtained with only half of the time points used (e.g., with GT_50%_) and averaged across all subjects/scans.
**Figure S11.** Comparison on the normalized mean error (NME, which is equivalent to a scaled cerebral blood flow [CBF] percentage error) of models without and with M_0_ included using the three‐dimensional (3D) U‐net and the pseudo‐3D SwinIR architectures. The NME was calculated in the whole brain. Although there were some differences between the models without and with M_0_ included at some averaging conditions, we did not observe the large increases of the bias when M_0_ was included as reported in Shou et al.^1^
**Table S1.** Similar to that shown in Table 2, except that the results were obtained using the pseudo‐3D (three‐slice) Swin‐IR transformer architecture. Trends like those using the three‐dimensional (3D) Unet architecture can be clearly observed.
**Table S2.** Like that shown in Table 3, except that the results were obtained using the pseudo‐3D (three‐slice) Swin‐IR transformer architecture. Trends like those using the three‐dimensional (3D) Unet architecture can be clearly observed.

## Data Availability

The *Python* code to train and test the 3D U‐net–based deep learning denoising models developed in this paper is available through the Open Science Framework (doi:10.17605/OSF.IO/GQ7ZW). The ASL Data sets 1–7 can be accessed from OpenNeuro (openneuro.org) with the OpenNeuro accession numbers listed in Table 1. The ASL Data Set 8 can be requested by contacting Dr. Nazem‐Zadeh.
