# Supplementary material for: Optimization of deep learning–based denoising for arterial spin labeling: Effects of averaging and training strategies
Source: Magn Reson Med. 2025 Aug 5;94(6):2715–31. doi: 10.1002/mrm.70013 (PMC12501684; doi:10.1002/mrm.70013)
Supplement: Supplementary file 1 — Figure S1. A schematic showing the three‐dimensional (3D) U‐net encoder‐decoder architecture used in this study. The numbers of the input channels are 1 and 2 for the models without and with M0 included, respectively. The deep learning operations and the dimensions of the tensors are color‐coded, as shown in the legends, and the channel numbers of the tensors after max pooling are shown at the top of the tensor blocks with slightly darker colors. BN, batch normalization; CONV, convolution; ReLU, rectified linear unit. Figure S2. An example showing the deep learning (DL)–denoising processing was able to suppress artifacts throughout the images which were likely caused by motion. Figure S3. Example showing that while the noise and some minor motion artifacts were satisfactorily suppressed by deep learning (DL) noising, the flow‐related artifacts (i.e., intravascular ASL signals) were faithfully preserved. Figure S4. Another example showing the deep learning (DL)–based denoising performance in a low–signal‐to‐noise ratio (SNR) scan. Some regional biases were observed when the DL models were trained and tested with low Nav values due to low SNR. Figure S5. Like that shown in Figure 4, except that the results were obtained with the pseudo‐3D (three‐slice) SwinIR transformer architecture. Trends like those using the 3D‐Unet architecture can be clearly observed. 3D, three‐dimensional. Figure S6. Like that shown in Figure 5, except that the results were obtained with the pseudo‐3D (three‐slice) SwinIR transformer architecture. Trends like those using the 3D U‐net architecture can be clearly observed, demonstrating that windowed averaging yielded superior performance than interleaved averaging overall. 3D, three‐dimensional. Figure S7. Like that shown in Figure 7, except that the results were obtained with the pseudo‐3D SwinIR transformer architecture and with all the time points used (e.g., with GT100%) and averaged across all subjects/scans. 3D, three‐dimensional. Figure S [file MRM-94-2715-s001.docx]

**Supporting Information**

**Supporting Information Table S1**. Similar to that shown in **Table 2**, except that the results were obtained using the pseudo-3D (3-slice) Swin-IR transformer architecture. Trends similar to those using the 3D-Unet architecture can be clearly observed.

|  | | **Mean SSIM** | | | | | **Mean PSNR (dB)** | | | | | **Mean NMAE** | | | | |
| --- | --- | --- | --- | --- | --- | --- | --- | --- | --- | --- | --- | --- | --- | --- | --- | --- |
| **Ind. denoised** | | **N_av_test_** | | | | | **N_av_test_** | | | | | **N_av_test_** | | | | |
|  |  | 1 | 2 | 4 | 8 | 16 | 1 | 2 | 4 | 8 | 16 | 1 | 2 | 4 | 8 | 16 |
| **N_av_train_** | 1 | **0.9063** | 0.9132 | 0.9187 | 0.9231 | 0.9271 | **23.0615** | 23.7868 | 24.3988 | 24.8835 | 25.2467 | **0.0289** | **0.0269** | 0.0255 | 0.0245 | 0.0238 |
|  | 2 | 0.9008 | **0.9141** | 0.9235 | 0.9302 | 0.9359 | 22.6619 | **23.8225** | 24.7793 | 25.5401 | 26.1678 | 0.0306 | 0.0270 | **0.0245** | 0.0228 | 0.0216 |
|  | 4 | 0.8844 | 0.9078 | **0.9257** | 0.9382 | 0.9476 | 21.3710 | 23.2759 | **24.9720** | 26.4096 | 27.7070 | 0.0374 | 0.0297 | 0.0246 | **0.0212** | 0.0188 |
|  | 8 | 0.8564 | 0.8889 | 0.9185 | **0.9417** | 0.9595 | 18.9838 | 21.5266 | 24.0810 | **26.4987** | 29.0454 | 0.0518 | 0.0377 | 0.0280 | 0.0214 | 0.0164 |
|  | 16 | 0.8215 | 0.8586 | 0.8963 | 0.9311 | **0.9641** | 16.0310 | 19.0375 | 22.2191 | 25.5006 | **29.6552** | 0.0784 | 0.0540 | 0.0371 | 0.0253 | **0.0156** |
| **After post-av.** | | **N_av_test_** | | | | | **N_av_test_** | | | | | **N_av_test_** | | | | |
|  |  | 1 | 2 | 4 | 8 | 16 | 1 | 2 | 4 | 8 | 16 | 1 | 2 | 4 | 8 | 16 |
| **N_av_train_** | 1 | 0.9189 | 0.9222 | 0.9251 | 0.9273 | 0.9292 | 24.5010 | 24.8807 | 25.1664 | 25.3530 | 25.4482 | 0.0257 | 0.0247 | 0.0239 | 0.0235 | 0.0233 |
|  | 2 | 0.9261 | 0.9302 | 0.9334 | 0.9361 | 0.9387 | 25.1507 | 25.6680 | 26.0503 | 26.3238 | 26.5168 | 0.0238 | 0.0228 | 0.0219 | 0.0213 | 0.0208 |
|  | 4 | 0.9408 | 0.9446 | 0.9474 | 0.9500 | 0.9526 | 26.4967 | 27.2233 | 27.7988 | 28.2451 | 28.5976 | 0.0208 | 0.0196 | 0.0187 | 0.0179 | 0.0173 |
|  | 8 | 0.9591 | 0.9635 | 0.9664 | 0.9684 | 0.9703 | 28.4709 | 29.5512 | 30.4318 | 31.0403 | 31.5319 | 0.0169 | 0.0154 | 0.0144 | 0.0137 | 0.0130 |
|  | 16 | **0.9838** | **0.9852** | **0.9867** | **0.9882** | **0.9895** | **34.7224** | **35.7439** | **36.5577** | **37.1710** | **37.6029** | **0.0094** | **0.0086** | **0.0079** | **0.0072** | **0.0066** |

**Supporting Information Table S2.** Similar to that shown in **Table 3**, except that the results were obtained using the pseudo-3D (3-slice) Swin-IR transformer architecture. Trends similar to those using the 3D-Unet architecture can be clearly observed.

|  | | **Mean SSIM (N_av_train_)** | | | | | **Mean PSNR (N_av_train_)** | | | | | **Mean NMAE (N_av_train_)** | | | | |
| --- | --- | --- | --- | --- | --- | --- | --- | --- | --- | --- | --- | --- | --- | --- | --- | --- |
|  |  | **Averaging method (N_av_pre_:N_av_post_)** | | | | | **Averaging method (N_av_pre_:N_av_post_)** | | | | | **Averaging method (N_av_pre_:N_av_post_)** | | | | |
|  |  | 1:N_tp_ | 2:(N_tp_/2) | 4:(N_tp_/4) | 8:(N_tp_/8) | 16:(N_tp_/16) | 1:N_tp_ | 2:(N_tp_/2) | 4:(N_tp_/4) | 8:(N_tp_/8) | 16:(N_tp_/16) | 1:N_tp_ | 2:(N_tp_/2) | 4:(N_tp_/4) | 8:(N_tp_/8) | 16:(N_tp_/16) |
| **N_tp_ available** | 1 | **0.9063**  **(1)** |  |  |  |  | **23.0615**  **(1)** |  |  |  |  | **0.02889**  **(1)** |  |  |  |  |
|  | 2 | 0.9128  (2) | **0.9141**  **(2)** |  |  |  | 23.7393  (2) | **23.8225**  **(2)** |  |  |  | 0.02731  (2) | **0.02694**  **(1)** |  |  |  |
|  | 4 | 0.9230  (4) | 0.9250  (4) | **0.9257**  **(4)** |  |  | 24.6874  (4) | 24.9233  (4) | **24.9720**  **(4)** |  |  | 0.02539  (4) | 0.02479  (4) | **0.02452**  **(2)** |  |  |
|  | 8 | 0.9377  (8) | 0.9405  (8) | 0.9417  (8) | **0.9417**  **(8)** |  | 25.9240  (8) | 26.3196  (8) | **26.5067**  **(8)** | 26.4987  (8) |  | 0.02264  (8) | 0.02178  (8) | 0.02140  (8) | **0.02123**  **(4)** |  |
|  | 16 | 0.9613  (16) | 0.9625  (16) | 0.9635  (16) | 0.9640  (16) | **0.9641**  **(16)** | 29.3790  (16) | 29.5900  (16) | 29.6824  (16) | **29.6895**  **(16)** | 29.6552  (16) | 0.01651  (16) | 0.01608  (16) | 0.01576  (16) | **0.01562**  **(16)** | 0.01564  (16) |


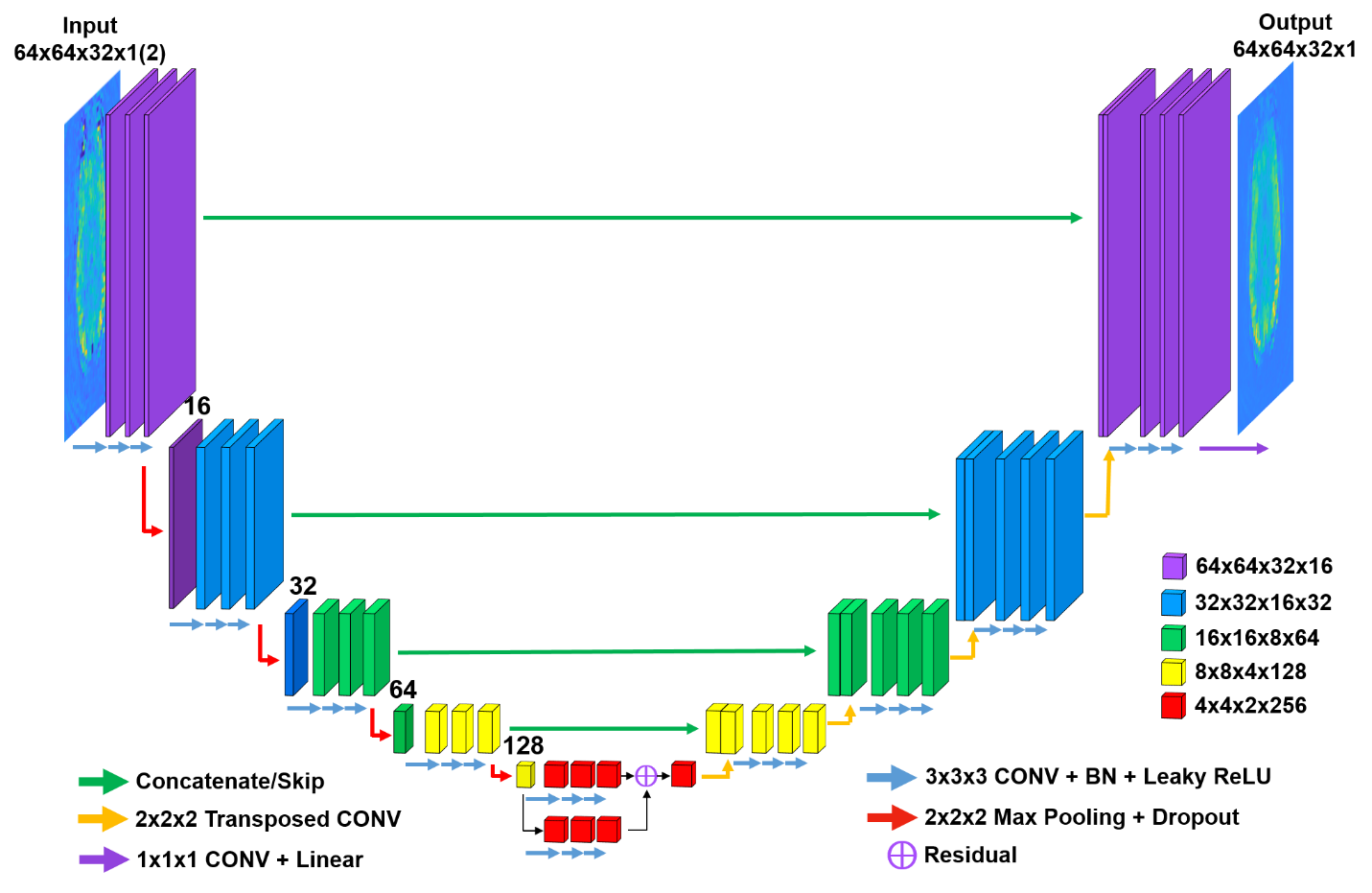


**Supporting Information Figure S1.** A schematic showing the 3D U-net encoder-decoder architecture used in this study. The numbers of the input channels are 1 and 2 for the models without and with M_0_ included, respectively. The deep learning operations and the dimensions of the tensors are color-coded as shown in the legends; and the channel numbers of the tensors after max pooling are shown at the top of the tensor blocks with slightly darker colors. CONV: convolution; BN: batch normalization; ReLU: rectified linear unit.


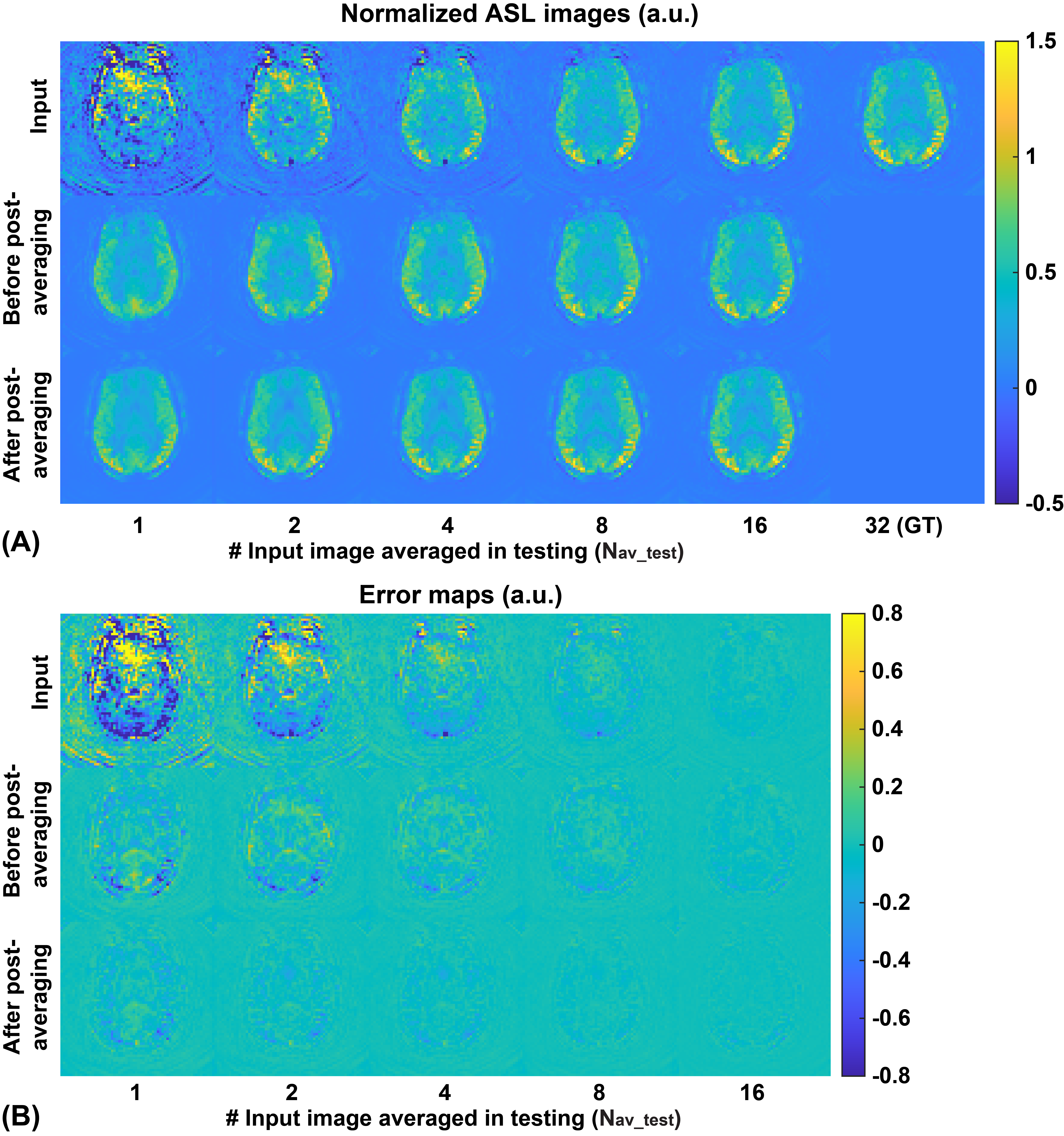


**Supporting Information Figure S2.** An example showing the DL-denoising processing was able to suppress artifacts throughout the images which were likely caused by motion.


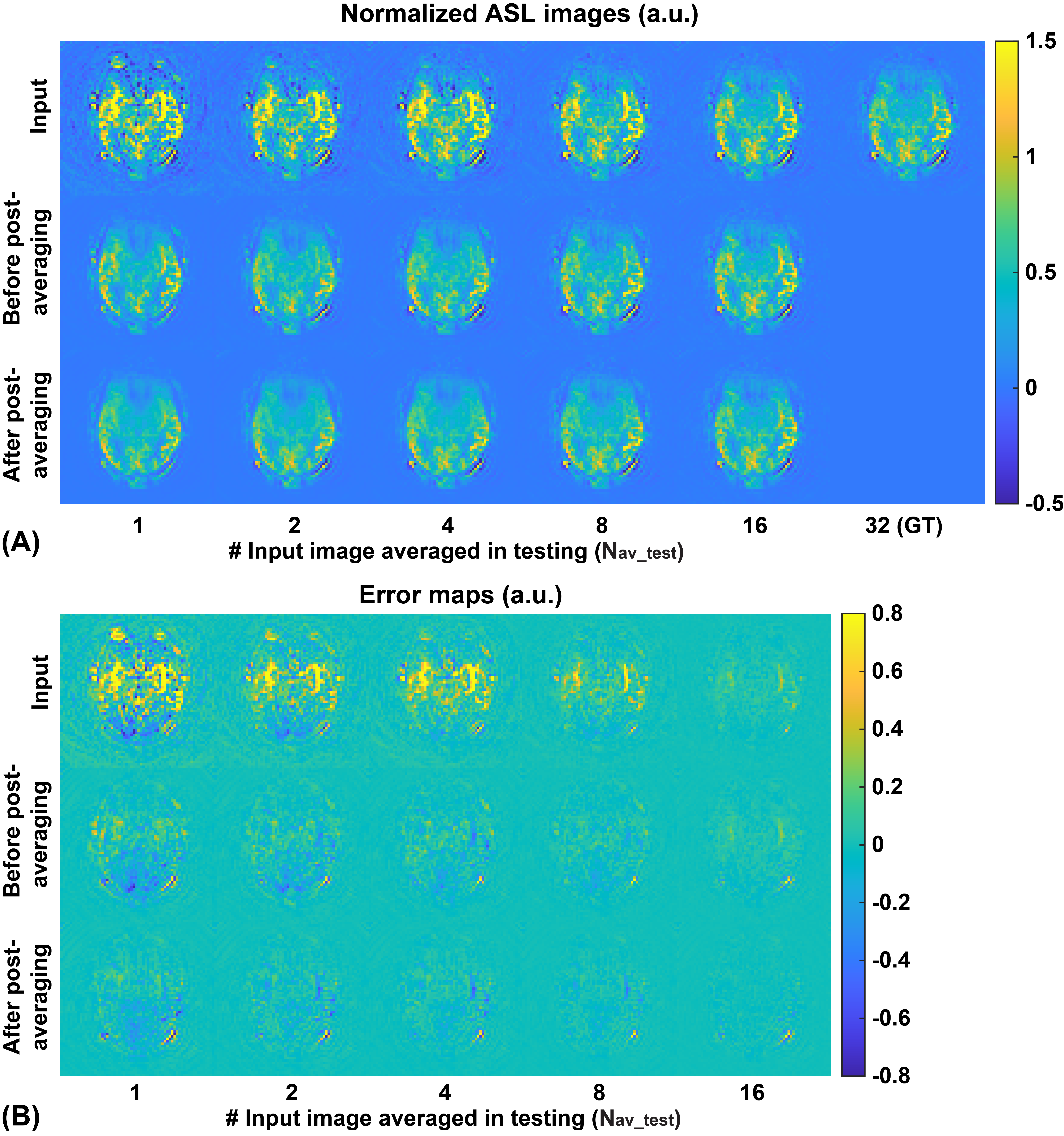


**Supporting Information Figure S3.** An example showing that while the noise and some minor motion artifacts were satisfactorily suppressed by DL noising, the flow-related artifacts, i.e. intravascular ASL signals, were faithfully preserved.


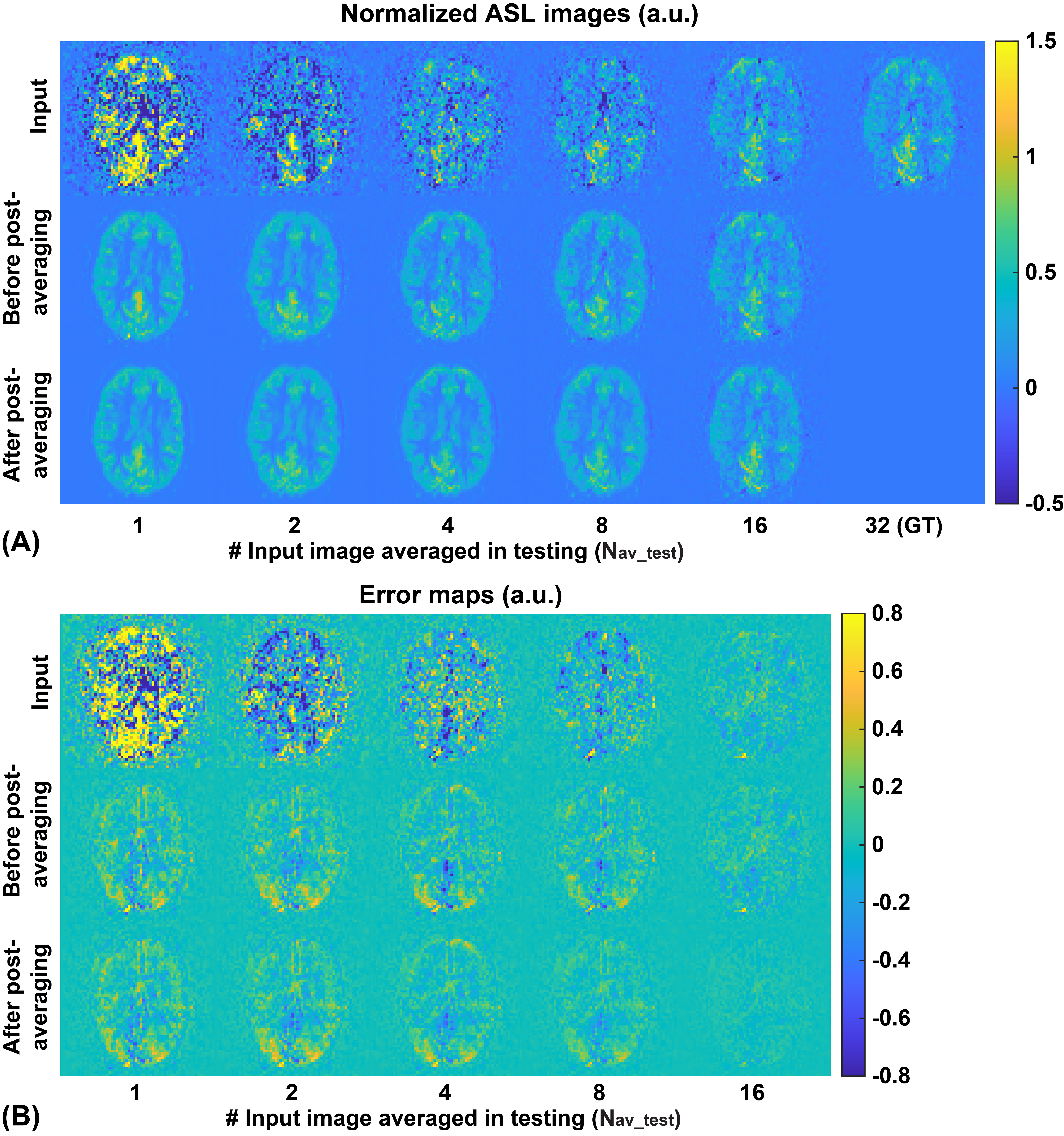


**Supporting Information Figure S4.** Another example showing the DL-based denoising performance in a low-SNR scan. Some regional biases were observed when the DL models were trained and tested with low N_av_ values due to low SNR.

**
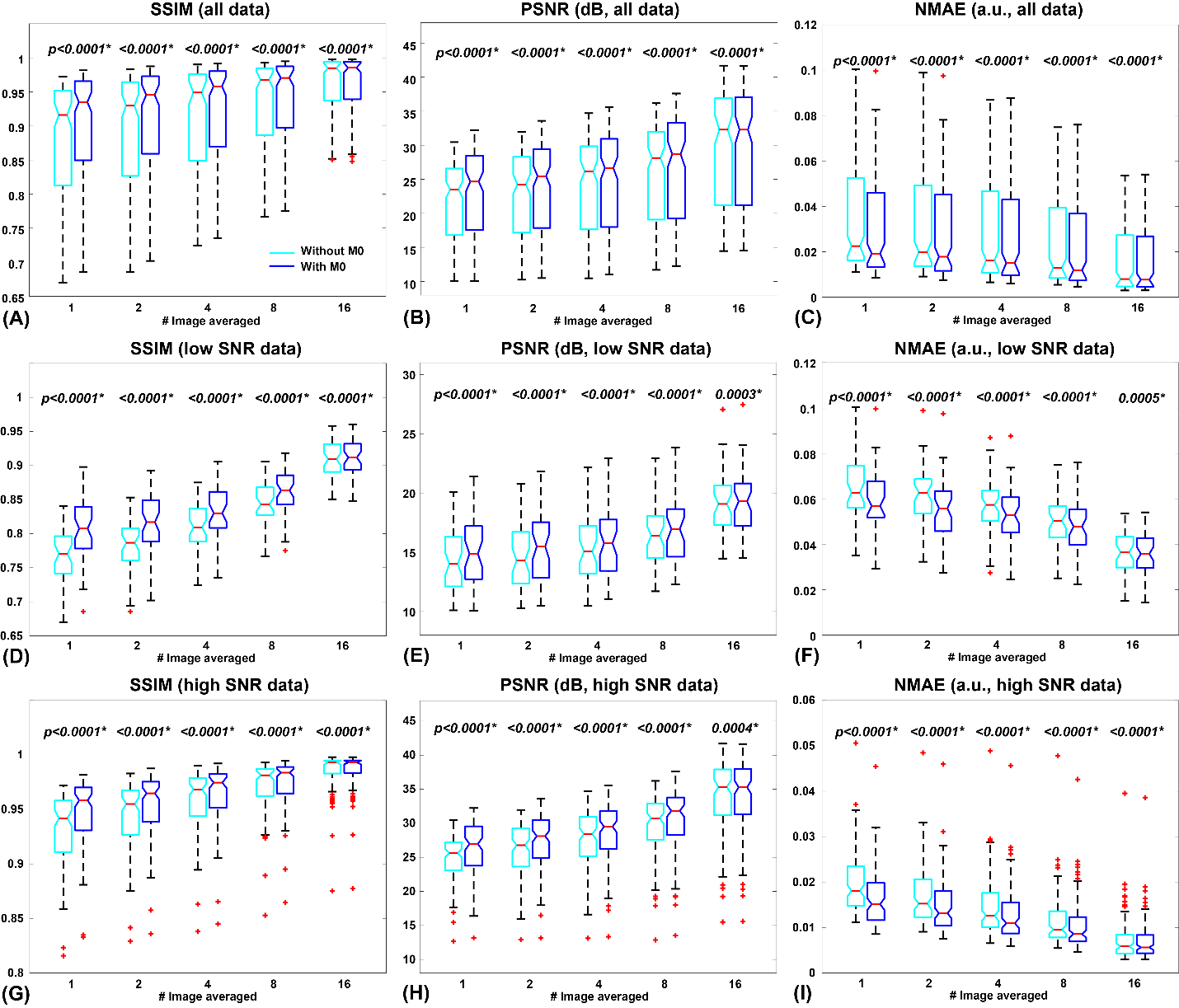
**

**Supporting Information Figure S5.** Similar to that shown in **Figure 4**, except that the results were obtained with the pseudo-3D (3-slice) SwinIR transformer architecture. Trends similar to those using the 3D-Unet architecture can be clearly observed.

**
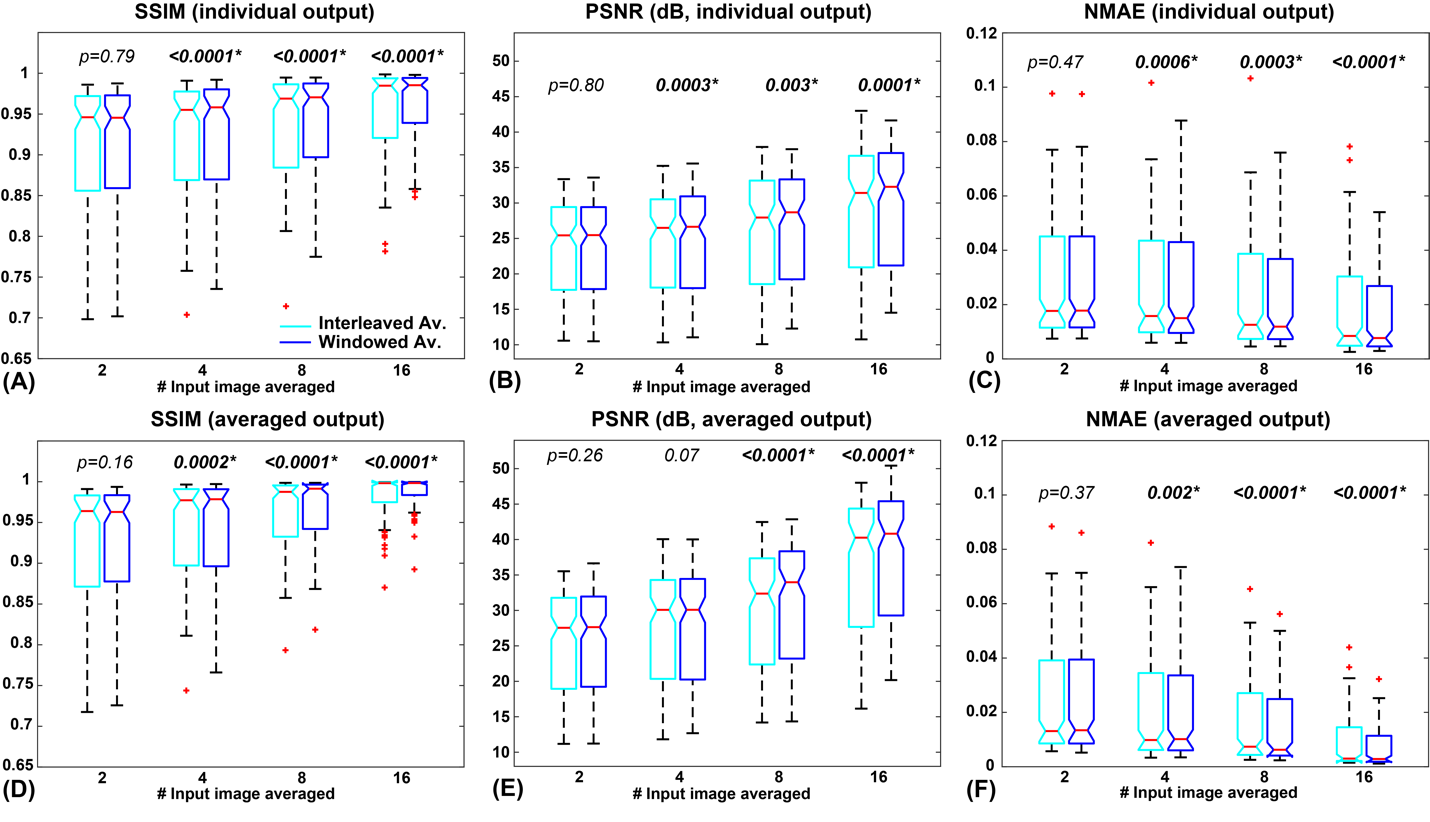
**

**Supporting Information Figure S6.** Similar to that shown in **Figure 5**, except that the results were obtained with the pseudo-3D (3-slice) SwinIR transformer architecture. Trends similar to those using the 3D U-net architecture can be clearly observed, demonstrating that windowed averaging yielded superior performance than interleaved averaging overall.


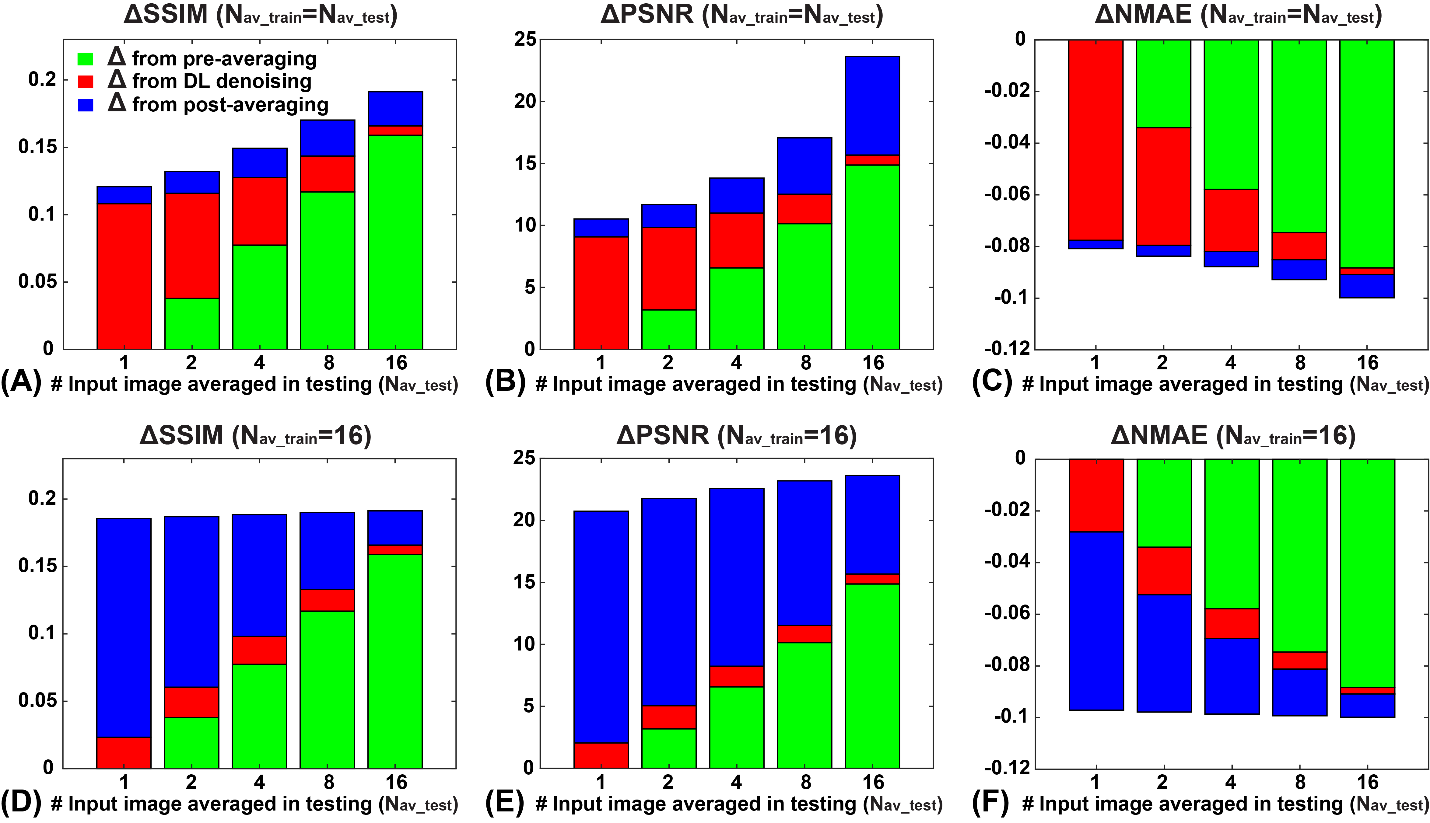


**Supporting Information Figure S7.** Similar to that shown in **Figure 7**, except that the results were obtained with the pseudo-3D SwinIR transformer architecture, and with all the time points used (e.g. with GT_100%_) and averaged across all subjects/scans.


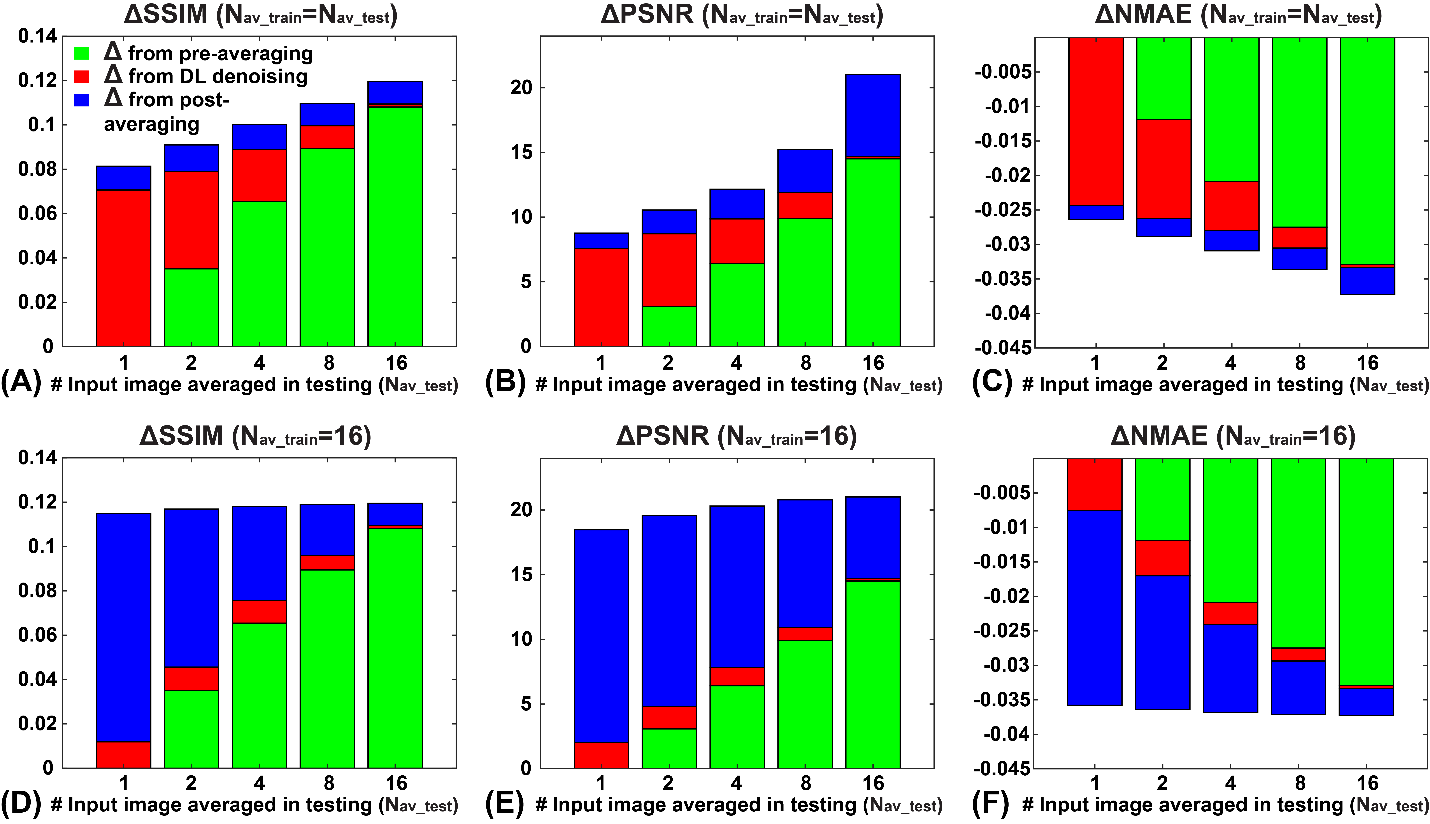


**Supporting Information Figure S8.** Similar to that shown in **Figure 7**, except that the results were obtained with all the time points used (e.g. with GT_100%_) and averaged across the high-SNR scans only.


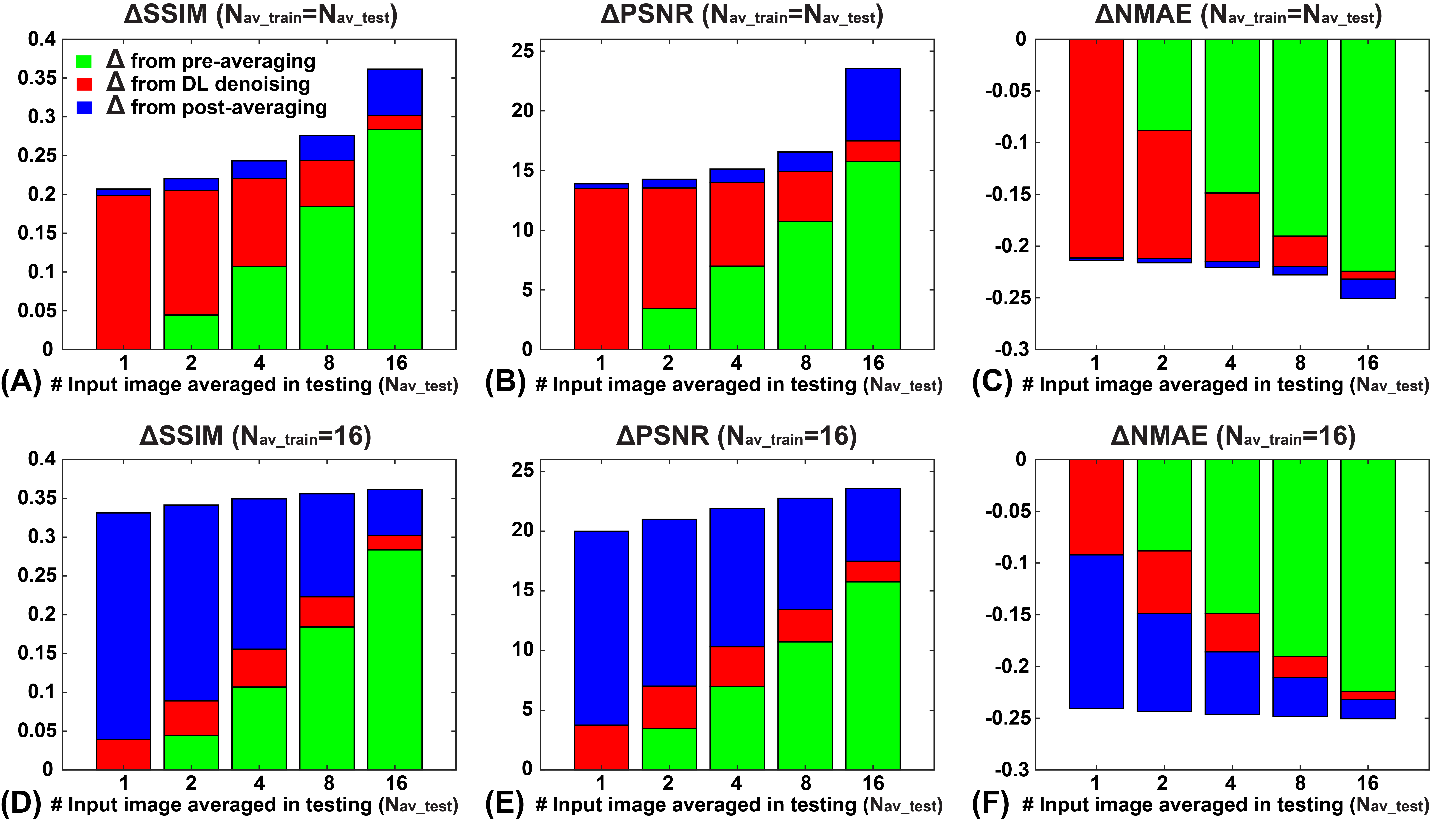


**Supporting Information Figure S9.** Similar to that shown in **Figure 7**, except that the results were obtained with all the time points used (e.g. with GT_100%_) and averaged across the low-SNR scans only.


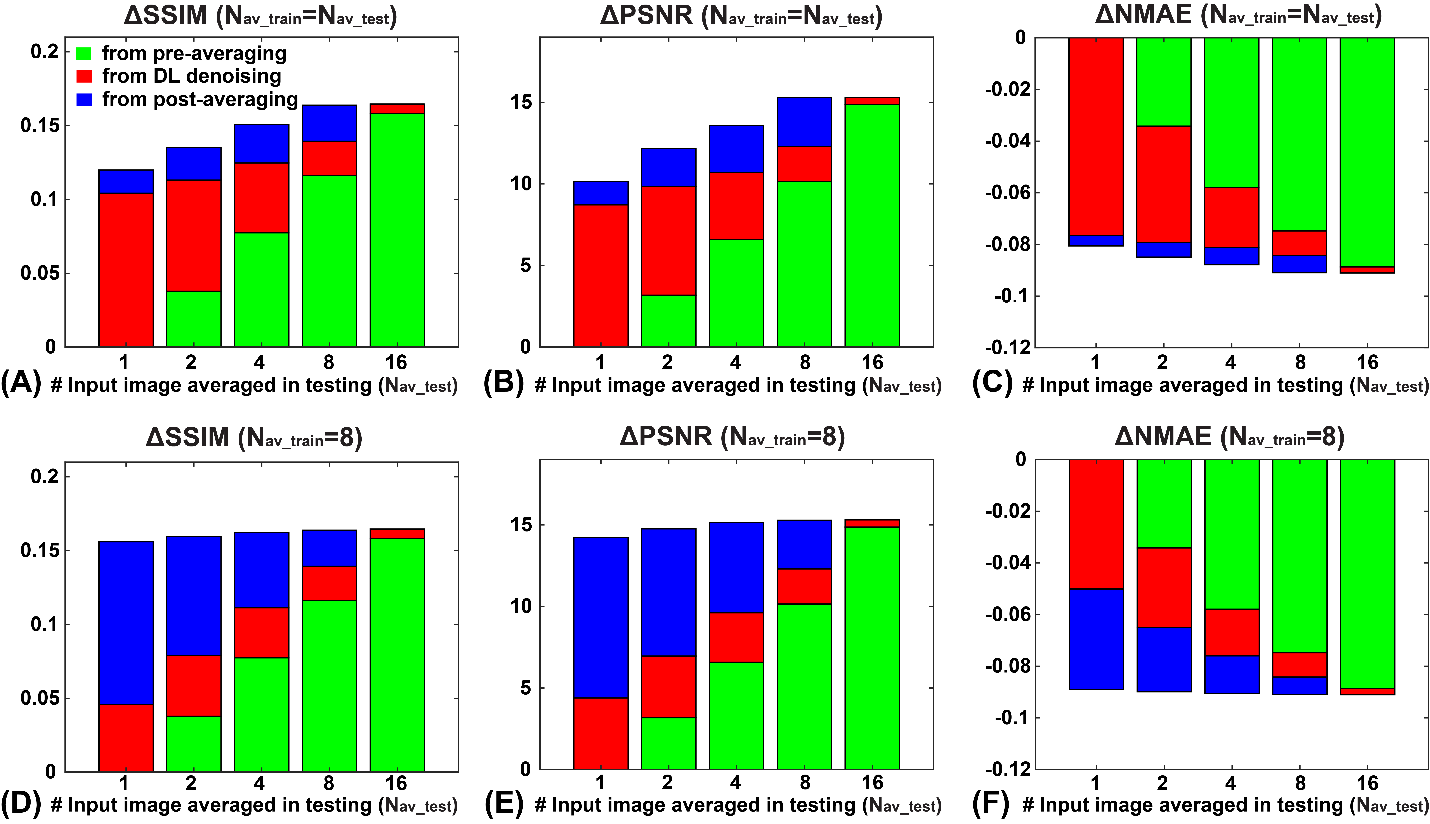


**Supporting Information Figure S10.** Similar to that shown in **Figure 7**, except that the results were obtained with only half of the time points used (e.g. with GT_50%_) and averaged across all subjects/scans.


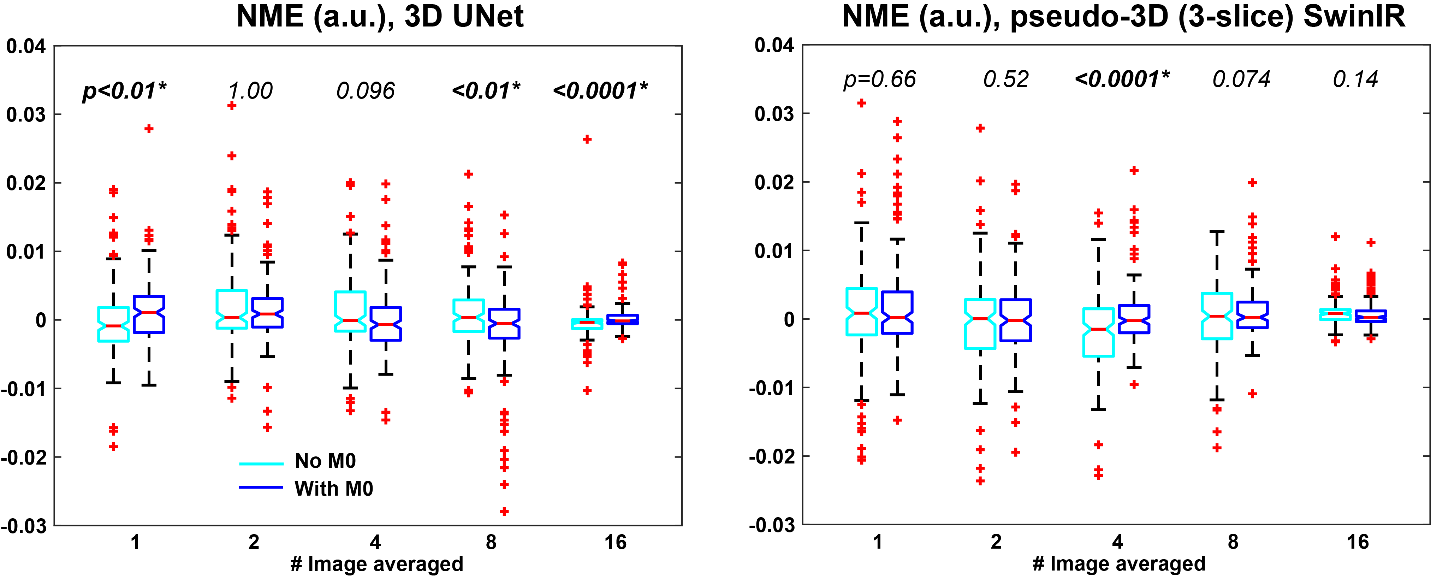


**Supporting Information Figure S11.** Comparison on the normalized mean error (NME, which is equivalent to a scaled CBF percentage error) of models without and with M_0_ included using the 3D U-net and the pseudo-3D SwinIR architectures. The NME was calculated in the whole brain. Though there were some differences between the models without and with M_0_ included at some averaging conditions, we did not observe the large increases of the bias when M_0_ was included as reported in Shou et al ^1^.

1. Shou Q, Zhao C, Shao X, Jann K, Kim H, Helmer KG, et al. Transformer-based deep learning denoising of single and multi-delay 3D arterial spin labeling. Magn Reson Med. 2024;91(2):803-18.
